# Protective Role of Cepharanthine Against Equid Herpesvirus Type 8 Through AMPK and Nrf2/HO-1 Pathway Activation

**DOI:** 10.3390/v16111765

**Published:** 2024-11-12

**Authors:** Shuwen Li, Liangliang Li, Yijia Sun, Muhammad Zahoor Khan, Yue Yu, Lian Ruan, Li Chen, Juan Zhao, Junchi Jia, Yubao Li, Changfa Wang, Tongtong Wang

**Affiliations:** 1 College of Agricultural Science and Engineering, Liaocheng University, Liaocheng 252000, China; 2 College of Veterinary Medicine, Shanxi Agricultural University, Taigu, Jinzhong 030801, China

**Keywords:** cepharanthine, oxidative stress, EqHV-8, antiviral activity, AMPK/Nrf2/HO-1 signaling pathway

## Abstract

Equid herpesvirus type 8 (EqHV-8) is known to cause respiratory disease and miscarriage in horses and donkeys, which is a major problem for the equine farming industry. However, there are currently limited vaccines or drugs available to effectively treat EqHV-8 infection. Therefore, it is crucial to develop new antiviral approaches to prevent potential pandemics caused by EqHV-8. This study evaluates the antiviral and antioxidant effects of cepharanthine against EqHV-8 by employing both in vitro assays and in vivo mouse models to assess its therapeutic efficacy. To assess the effectiveness of cepharanthine against EqHV-8, we conducted experiments using NBL-6 and RK-13 cells. Additionally, we developed a mouse model to validate cepharanthine’s effectiveness against EqHV-8. In our in vitro experiments, we assessed the cepharanthine’s ability to inhibit infection caused by EqHV-8 in NBL-6 and RK-13 cells. Our results demonstrated that cepharanthine has a dose-dependent inhibitory effect, indicating that it possesses anti-EqHV-8 properties at the cellular level. Moreover, we investigated the mechanism through which cepharanthine exerts its protective effects. It was observed that cepharanthine effectively reduces the oxidative stress induced by EqHV-8 by activating the AMPK and Nrf2/HO-1 signaling pathways. Furthermore, when administered to EqHV-8 infected mice, cepharanthine significantly improved lung tissue pathology and reduced oxidative stress. The findings presented herein collectively highlight cepharanthine as a promising candidate for combating EqHV-8 infections.

## 1. Introduction

Herpesviruses are a diverse group of viruses that can be classified into three subtypes: alpha herpesviruses, beta herpesviruses, and gamma herpesviruses. These viruses exhibit a broad host range, infecting various species, including mammals, birds, fish, and reptiles [1,2,3]. In the context of equine herpesviruses (EHVs), nine subtypes have been identified, with five of them belonging to the alpha herpesviruses group. Horses are the primary hosts for equine herpesviruses EHV-1 through EHV-5, while donkeys are the natural hosts of EHV-6 to EHV-8, also referred to as AHV-1 to AHV-3 [4,5].

EHV-8 is a double-stranded DNA virus with a genome size of 150 kb and encodes 76 open-reading frames [6]. It was first isolated in donkeys in Australia in 1988 [7]. Since then, it has been found in horse and donkey populations in China and Israel [8,9]. Notably, China has experienced a notable increase in respiratory diseases and miscarriages among donkeys, resulting in significant economic losses. These issues have been linked to infections caused by EqHV-8 [10]. Studies have shown that EqHV-8 can cause severe respiratory disease, neurological disorders in equids, and miscarriages in mares [11,12,13]. However, the development of effective vaccines and treatments against EqHV-8 infections remains a challenge.

Cepharanthine, a natural bisbenzylisoquinoline (BBIQ) alkaloid extracted from Stephania cepharantha Hayata, has been employed in Japan since 1951 for the treatment of radiation-induced leukopenia and alopecia pityrodes [14]. Recent research has highlighted its anti-inflammatory, antioxidative, and antiviral properties [15]. For instance, studies have shown that cepharanthine can reduce cerebral ischemia/reperfusion injury in mice through its anti-inflammatory and antioxidant effects [16]. Additionally, it has been demonstrated that cepharanthine exerts protective effects against lipopolysaccharide (LPS)-induced oxidative stress and inflammatory responses in macrophages by activating the AMP-activated protein kinase (AMPK)-α1/AKT/GSK-3β/NRF2 signaling pathway [17]. Furthermore, cepharanthine has been found to inhibit the replication of herpes simplex virus type 1 (HSV-1) by modulating the PI3K/Akt and p38 MAPK signaling pathways [18], and it also inhibits severe acute respiratory syndrome coronavirus 2 (SARS-CoV-2) infection by regulating the PI3K/Akt, relaxin, VEGF, and HIF-1 signaling pathways [19]. However, the specific antiviral activities of cepharanthine against EqHV-8 and the underlying mechanisms remain poorly understood.

In our current study, we aimed to investigate the effectiveness of cepharanthine against infection of EqHV-8 and explore the underlying molecular mechanisms involved. Our findings show that cepharanthine significantly inhibits EqHV-8 replication in EqHV-8-susceptible RK-13 and NBL-6 cells, with this inhibition occurring at the stages of cell adsorption and internalization. Importantly, our results suggest that the anti-EqHV-8 effect of cepharanthine is associated with the activation of AMPK and Nrf2/HO-1 pathways. Taken together, our study provides compelling evidence that cepharanthine holds promise as a potential therapeutic agent for the control of EqHV-8 infections.

## 2. Materials and Methods

### 2.1. Cells, Viruses, and Reagents 

Rabbit kidney cells (RK-13) were purchased from the China Center for Type Culture Collection and cultured in MEM (Life Technologies Corporation, Waltham, MA, USA) supplemented with 10% fetal bovine serum (FBS, Gibco, GrandIsland, NE, USA) and 1% penicillin–streptomycin (Solarbio, Beijing, China) at 37 °C and 5% CO_2_ and E. Derm cells (NBL-6) were the American Type Culture Collection and maintained in Modified Eagle Medium (MEM) supplemented with 10% FBS (Gibco, GrandIsland, NE, USA) and 1% penicillin–streptomycin. The EHV-8 strains used for present study were as follows: SDLC66 (GenBank: MW816102.1), SD2020113 (GenBank: MW822570.1), and donkey/Shandong/10/2021 (GenBank: OL856098.1). Cepharanthine was purchased from Shandong SparkJade Biotechnology Co., Ltd. (Jinan, China) and dissolved in dimethyl sulfoxide (DMSO) (Servicebio, Wuhan, China), BR ELISA kit was purchased from Elabscience (E-EL-0076, Wuhan, China). Endogenous carbon monoxide test kit was purchased from Shanghai Yiji Industrial Co., Ltd. (A101-2-1, Shanghai, China). Intracellular Iron Colorimetric Assay Kit was purchased from Applygen Technologies Inc. (E1042, Beijing, China).

### 2.2. Cell Viability Analysis

Cepharanthine’s cytotoxicity was assessed using the cell counting kit-8 (CCK-8) assay (Beyotime, Nanjing, China), following the protocol described previously [20]. RK-13 and NBL-6 cells were seeded into 96-well plates (1 × 10^4^ cells/well) and treated with different concentrations of cepharanthine (0, 1.25, 2.5, 5, 10, 15, and 30 μM) for 24 h. After that, 10 µL of CCK-8 reagent was added to each well, which contained 100 µL of medium, and incubated at 37 °C for 2 h, following the manufacturer’s instructions. The viable cell count was assessed through absorbance measurements at 450 nm by microplate reader (BioTek, Winooski, VT, USA). Cell survival was calculated as follows: cell survival rate (%) = [OD (sample) − OD (blank)/OD (control) − OD (blank)] × 100%.

### 2.3. Antiviral Activity of Cepharanthine

RK-13 and NBL-6 cells were seeded into 6-well plates and cultured until they were 80–90% confluent. Subsequently, they were treated with various cepharanthine concentrations (1.25, 2.5, and 5 μM) or DMSO (control) for 2 h, followed by EqHV-8 SDLC66 infection (MOI = 0.1) for 1 h. The treatment medium was then replaced with 3% FBS in MEM containing cepharanthine, and the cells and cellular supernatant were collected for further Western blot and the 50% tissue culture infectious dose (TCID_50_) analysis. NBL-6 cells were transfected with siRNAs targeting AMPK, Nrf2, and HO-1 (siAMPK, siNrf2, and siHO-1) or negative control (siNC) for 12 h. Then, they were infected with EqHV-8 SDLC66 at MOI = 0.1. Finally, the cells were harvested at 24 hpi to assess EqHV-8 replication via Western blot analysis. 

### 2.4. Indirect Immunofluorescence Assay

Cepharanthine-treated EqHV-8-infected or normal RK-13 and NBL-6 cells were fixed in 75% ethanol (500 μL/well) for 30 min at 4 °C. The cells were washed and treated with anti-EqHV-8-positive serum prepared in our laboratory (1:1000 dilution; used as the primary antibody). The cells were then incubated with a secondary Rhodamine-conjugated goat anti-mouse IgG antibody at a dilution of 1:500. Following this, they were counterstained with 4′,6-diamidino-2-phenylindole (DAPI) and visualized using a DMi8 microsystems (Leica, Wetzlar, Germany). Mock-infected cells served as controls to determine background staining levels.

### 2.5. Western Blot

Cells were lysed using NP40 lysis buffer (Beyotime, Nanjing, China), and protein lysates were separated using 12% SDS-PAGE gels and transferred to polyvinylidene difluoride (PVDF) membranes. PVDF membranes were blocked with 5% non-fat dry milk for 1 h. Then, the membranes were incubated with primary antibodies (anti-EqHV-8 gD (Prepared by our laboratory), 1:200; anti-HO-1 (GB12104, Servicebio, Wuhan, China), 1:1000; anti-AMPK/p-AMPK (GB112669, Servicebio, Wuhan, China/ab133448, Abcam, Cambridge, UK), 1:1000; anti-Nrf2 (GB113808, Servicebio, Wuhan, China), 1:500; or anti-α-tubulin (T6199, Sigma, St. Louis, MO, USA), 1:1000). Subsequently, the membranes were washed with phosphate-buffered saline with Tween 20 (PBST) and then treated with an HRP-conjugated goat anti-mouse antibody as the secondary antibody. Finally, specific bands were visualized using the ECL reagent (Thermo Fisher, San Jose, CA, USA) on a ChemiDoc MP Imaging System (BioRad, Hercules, CA, USA). 

### 2.6. Virus Titration

The production of viral progeny was determined via titration, as previously described [12]. Briefly, RK-13 cells were cultured in 96-well plates and grown to 70–80% confluency. Subsequently, the viral supernatant was serially diluted 10-fold to generate eight dilutions, using 100 μL of diluent per well. The cells were cultured at 37 °C for 3–5 days, and the cytopathic effects were observed every day. The TCID_50_ was calculated using the Reed–Muench method.

### 2.7. Time-Course Analysis and Direct Inactivation Assay

To investigate which stage of the EqHV-8 life cycle is affected by cepharanthine, NBL-6 cells were inoculated in 12-well plates. Different treatment groups were established, and cells were treated with cepharanthine at the indicated concentration and with EqHV-8 at an MOI = 0.1. Pre-treated (M1), co-treated (M2), and post-treated (M3) cells were collected to assess viral replication using Western blot. 

For the inactivation assay, NBL-6 cells were seeded into 12-well plates once they reached 80% confluence. EqHV-8 SDLC66 was incubated with cepharanthine (5 µM) at 37 °C for 1 h at both 0.1 MOI and 0.5 MOI. The virus, after treatment, was then used to infect NBL-6 cells for 1 h. At 24 hpi, cells and supernatants were harvested to assess EqHV-8 replication through Western blot analysis and TCID_50_ assays.

### 2.8. RNA/DNA Extraction and Real-Time PCR Analysis

Total RNA was extracted from cells using TRIzol reagent (Sparkjade, Jian, China) and then reverse transcribed with the PrimeScript™ RT reagent Kit (Takara, Dalian, China) according to the manufacturer’s guidelines. Quantitative PCR (qPCR) was performed on a Step One Plus Real-Time PCR System, utilizing the SYBR™ Green PCR Master Mix (Genstar, Beijing, China) along with specific forward and reverse primers for the gD gene of EqHV-8. GAPDH mRNA was used as the internal control. The expression levels of the target gene were analyzed using the 2^−∆∆Ct^ method, as described in previous studies [21]. The primer sequences for qPCR are listed in Table 1.

To assess the viral copies of EqHV-8 in the supernatant, the gD fragment from the ORF72 gene was amplified using the ORF72-F and ORF72-R primers (Table 1) and cloned into the pMD18-T vector, generating the recombinant plasmid pMD18-T-gD for standard curve creation. Simultaneously, DNA was extracted from the viral supernatant using the DNA Viral Genome Extraction Kit (Solarbio, Beijing, China), and absolute quantification was carried out via qPCR, as outlined in earlier research [22]. The viral copies of EqHV-8 in the supernatant were subsequently determined using the standard curve.

### 2.9. Small-Interfering RNA Assays

All siRNAs used in the present study were synthesized by GenePharma Co., Ltd. (Shanghai, China) and are listed in Table 2. NBL-6 cells were pre-seeded into a 12-well plate overnight. The cells were treated with a mixture of Lipo6000™ Transfection Reagent (Beyotime Biotechnology, China) and 100 nmol siNC (negative control) or siAMPK/siNrf2/siHO-1 for 12 h and then treated with cepharanthine (5 μM) before EqHV-8 SDLC66 (MOI = 0.1) infection for 24 h. Finally, cells were harvested to analyze replication of EqHV-8 using Western blot and qPCR assays.

### 2.10. Detection of Superoxide Dismutase (SOD), Glutathione (GSH-PX), Reactive Oxygen Species (ROS), and Malonaldehyde (MDA)

A dichlorofluorescein (DCF) detection kit for ROS (Beyotime Biotechnology, Shanghai, China), MDA, SOD, and GSH-PX detection kits (Nanjing Jiancheng Bioengineering Institute, Nanjing, China) were utilized to examine the levels of SOD, GSH-PX, ROS, and MDA in NBL-6 cells following manufacturer’s instructions. A spark microplate reader (Tecan, Mennedorf, Switzerland) and DMi8 fluorescence microscope (Leica, Wetzlar, Germany) were used for the assessment of fluorescent intensity quantification and ROS generation, respectively. The levels of these indicators were normalized to protein concentrations measured using the Pierce^TM^ BCA Protein Assay Kit (Thermo Fisher, San Jose, CA, USA).

### 2.11. Inhibition of AMPK Activity

The inhibition of AMPK activity was determined after treatment with specific compounds. Each compound was diluted in a 10% DMSO solution, and 5 μL of this diluent was incorporated into a 50 μL reaction mixture, resulting in a final DMSO concentration of 1%. The reaction mixture included 40 mM Tris, 10 mM MgCl_2_, 0.1 mg/mL bovine serum albumin, 1 mM dithiothreitol (DTT), 10 μM ATP, 0.4 μg/mL AMPK, along with the AMPK substrate (0.1 mg/mL AMARA peptide and 100 μM AMP). The mixture was incubated at 30 °C for 40 min. Detection was carried out using a kinase detection kit from Beyotime (Haimen, Shanghai, China).

### 2.12. Determination of ATP or ADP Content

The levels of ATP, ADP, and AMP were measured following treatment of NBL-6 cells with various concentrations of cepharanthine for 24 h. After treatment, cells were collected and centrifuged at 13,680× *g* for 5 min, and both the cell pellets and supernatants were retained. The cell pellets were then resuspended in 200 μL of phosphate-buffered saline (PBS), and the total protein concentration was assessed using Pierce BCA Protein Assay Kit (Thermo Fisher, USA). The ATP content was measured using an ATP assay kit from Beyotime (Haimen, China), while the levels of ADP and AMP were analyzed using an enzyme-linked immunosorbent assay (ELISA) kit from Mlbio (Shanghai, China).

### 2.13. In Vivo Anti-EqHV-8 Assay 

Twenty 8-week-old SPF BALB/c male mice were obtained from the Laboratory Animal Center of Shandong Province (Jinan, China) and randomly divided into 4 groups (*n* = 5/group). Mice were intraperitoneally treated with cepharanthine (30 μmol/kg) or DMSO (0.5 mL/kg), as indicated, and then intranasally inoculated with EqHV-8 (1 × 10^5^ PFU/mice) or MEM under deep anesthesia (Zoletil 50; Virbac, Nice, France). The treatment regimen for each group is shown in Table 3. All mice were provided unrestricted access to food and water and were housed in a quarantine room to avoid cross-infection. The clinical signs and body weight of the mice were monitored daily. The mice were finally executed via cervical dislocation at 7 dpi, and lung tissue was collected to perform histopathologic analysis and evaluate viral replication. 

### 2.14. Histopathological Evaluation

The lung tissues of all mice were obtained and fixed with 10% formalin solution. The tissues were embedded in paraffin wax, sliced into 4 µm sections using a microtome (Leica, Germany), affixed onto slides, and then subjected to hematoxylin and eosin (HE) staining, as previously described [13]. The stained tissue samples were subsequently examined using a light microscope (Leica, Wetzlar, Germany).

### 2.15. Statistical Analysis

Data are presented as mean ± standard deviation (SD). GraphPad Prism 8.0 (San Diego, CA, USA) was used for statistical analyses. Differences between groups were assessed using the unpaired Student’s *t*-test. The significance levels were defined as follows: * *p* < 0.05, **, *p* < 0.01, and ***, *p* < 0.001.

## 3. Results

### 3.1. Cepharanthine Inhibits EqHV-8 in a Dose-Dependent Manner

**Figure 1:**
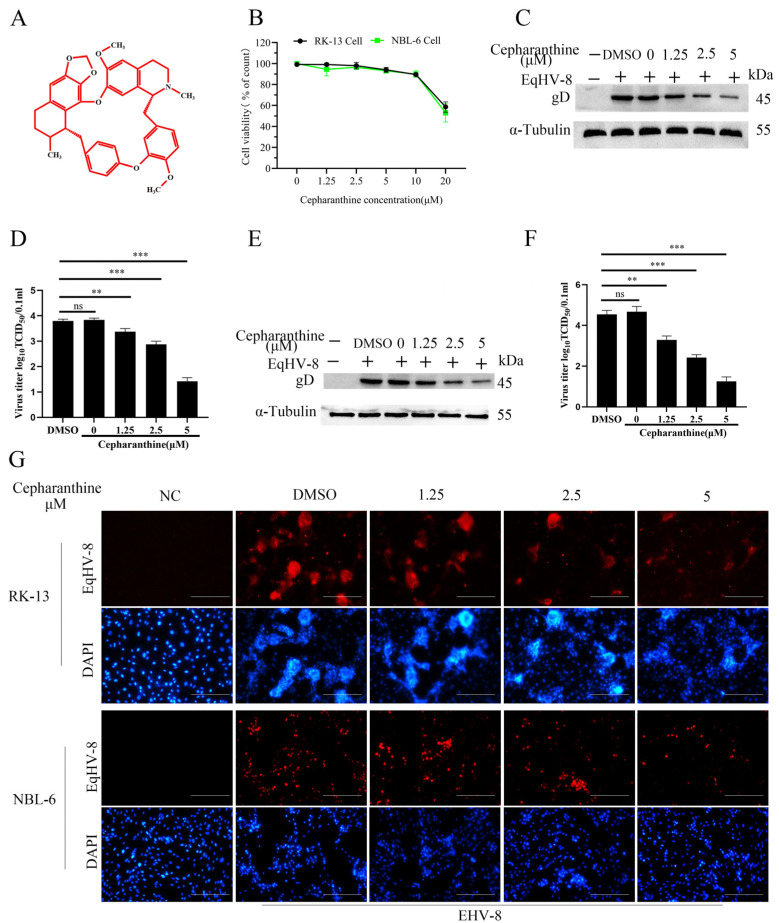
The effect of cepharanthine on the replication of EqHV-8 in susceptible cell lines. (**A**) Chemical structure of cepharanthine. (**B**) The cytotoxic effects of cepharanthine were evaluated in RK-13 and NBL-6 cell lines using a CCK-8 assay. Cell viability was expressed relative to control cells (which received no treatment), designated as 100%. The results represent data from three independent experiments. RK-13 (**C**,**D**) and NBL-6 (**E**,**F**) cells were treated with varying concentrations of cepharanthine (0, 1.25, 2.5, and 5 μM) for 2 h before being infected with EqHV-8 SDLC66 at a multiplicity of infection (MOI) of 0.1 for 1 h at 37 °C. Cells were harvested at 24 hpi to assess EqHV-8 replication via Western blotting and TCID_50_ assays. Additionally, an indirect immunofluorescence assay was conducted. Images of the EqHV-8 SDLC66-infected RK-13 and NBL-6 cells treated with cepharanthine were captured at 36 hpi. EqHV-8 proteins are depicted in red, while the nucleocapsid (stained with DAPI) appears in blue (**G**). Data are presented as mean values of normalized results ± standard deviation (indicated by error bars) derived from at least three independent experiments. ** *p* < 0.01; *** *p* < 0.001; ns represents not significant. Scale bar: 50 µm.

In our investigation, we first sought to elucidate the potential cytotoxicity of cepharanthine. The chemical structure of cepharanthine is illustrated in Figure 1A. To assess its impact on cell viability, we employed the CCK-8 assay, examining a range of cepharanthine concentrations on RK-13 and NBL-6 cells. Our findings reveal that, up to a concentration of 5 μM, cepharanthine does not exert any discernible influence on cell survival in both NBL-6 and RK-13 cells (Figure 1B). To further test the anti-EqHV-8 activity of cepharanthine, we conducted a series of experiments. Initially, NBL-6 and RK-13 cells were pre-treated with varying concentrations of cepharanthine for 2 h. Furthermore, these cells were subjected to EqHV-8 SDLC66 infection (MOI = 0.1) for 1 h. Following incubation, both cells and cellular supernatant were separated to assess EqHV-8 replication, employing Western blot and TCID_50_ assays. Remarkably, our investigations in RK-13 cells indicated that cepharanthine effectively inhibited the protein expression of EqHV-8 glycoprotein D (gD) (Figure 1C). Additionally, the production of viral progeny, as measured by TCID_50_, exhibited significant reduction at concentrations of 1.25 µM, 2.5 µM, and 5 µM of cepharanthine, compared to DMSO-treated cells (Figure 1D). In NBL-6 cells, similar results were observed (Figure 1E,F). Concurrently, indirect immunofluorescence assays confirmed these findings, demonstrating that cepharanthine effectively reduces infection caused by EqHV-8 infection in susceptible NBL-6 and RK-13 cells (Figure 1G).

### 3.2. Cepharanthine Exerts Antiviral Effect Against EqHV-8 Strains with Different Doses and Strains

**Figure 2:**
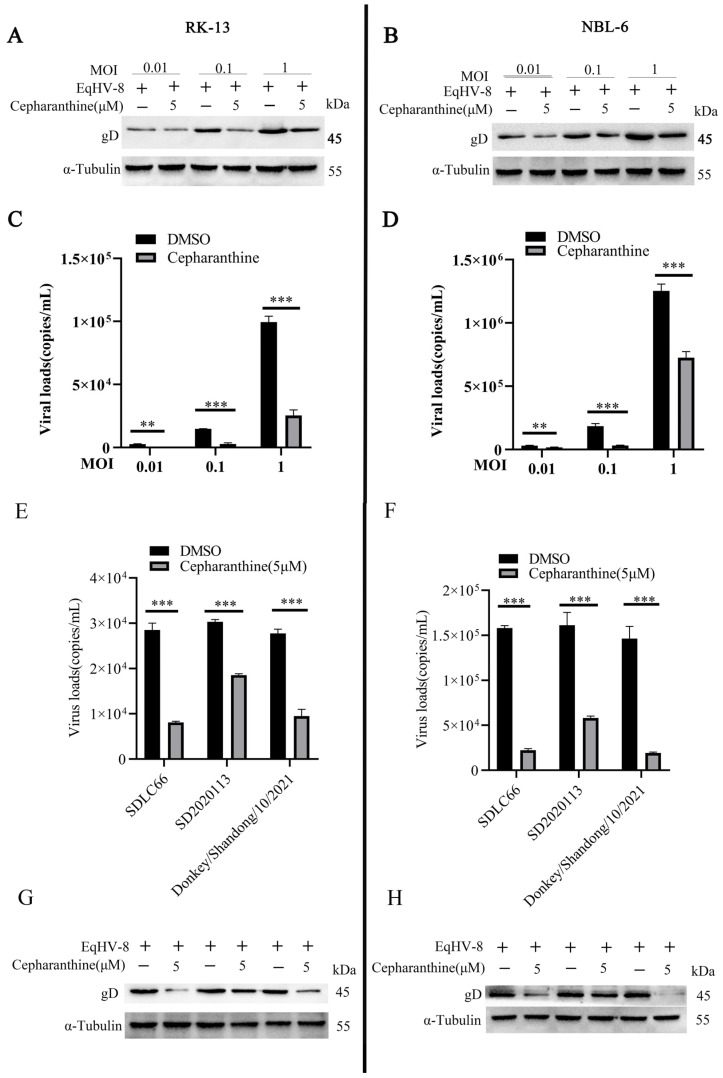
Cepharanthine demonstrates anti-EqHV-8 activity across multiple doses and strains. To investigate the effects of cepharanthine on EqHV-8, EqHV-8-susceptible cells were pretreated with cepharanthine at a concentration of 5 μM for 2 h. Following pretreatment, the cells were infected with EqHV-8 strain SDLC66 at MOI of 0.01, 0.1, and 1 for 1 h at 37 °C. Subsequently, the cells were washed with PBS, and the culture medium was replaced with a 3% FBS MEM containing 5 μM cepharanthine. At 24 hpi, both the cells and the cellular supernatants were collected for analysis of EqHV-8 infection. The expression of the gD protein was evaluated using Western blotting in RK-13 (**A**) and NBL-6 (**B**) cells. Additionally, the viral DNA copies were quantified using qPCR in RK-13 (**C**) and NBL-6 (**D**) cells. In further experiments, the EqHV-8-susceptible cells were again pretreated with cepharanthine (5 μM) for 2 h and subsequently infected with EqHV-8 strains SDLC66, SD2020113, or Donkey/Shandong/10/2021 at an MOI of 0.1. Following the infection, the cells were washed with PBS and incubated in a 3% FBS MEM supplemented with 5 μM cepharanthine. Progeny virus production and gD protein expression were assessed through qPCR and Western blot assays in RK-13 (**E**,**F**) cells at 24 hpi, with similar procedures applied to NBL-6 cells (**G**,**H**). α-Tubulin served as the loading control. Data are expressed as means of normalized values ± standard deviations (error bars) from a minimum of three independent experiments. Statistical significance was determined with ** *p* < 0.01; *** *p* < 0.001.

To investigate whether cepharanthine’s inhibitory effect extends to various titers of EqHV-8, we pre-treated NBL-6 and RK-13 cells with 5 μM of cepharanthine. Then, we incubated the cells with EqHV-8 SDLC66 at different MOIs (0.01, 0.1, and 1). We collected cells and cellular supernatants to assess EqHV-8 replication using qPCR and Western blot assays. Our results unequivocally indicate that cepharanthine effectively reduces gD protein expression and inhibits progeny virus production in both RK-13 (Figure 2A,B) and NBL-6 cells (Figure 2C,D). These findings suggest that cepharanthine could suppress EqHV-8 infection across a wide range of viral loads.

To further test whether cepharanthine could inhibit other strains of EqHV-8, we pre-treated NBL-6 and RK-13 cells with 5 μM of cepharanthine for 2 h. Then, we incubated the cells with EqHV-8 strains: SDLC66, SD2020113, and donkey/2021 (MOI = 0.1) for 1 h, respectively. Furthermore, we replaced the culture medium with 3% FBS in MEM consisting of cepharanthine and collected samples for EqHV-8 replication assessment using Western blot and qPCR assays. Our results showed that cepharanthine significantly reduces progeny virus copies number and lowers the level of gD protein expression in RK-13 (Figure 2E,F) and NBL-6 (Figure 2G,H) cells compared to the DMSO treatment group. This compelling evidence strongly suggests that cepharanthine exerts inhibitory effects against a broad spectrum of EqHV-8 strains. 

### 3.3. Cepharanthine Shows Antiviral Activity in the Early Stages of EqHV-8 Infection 

**Figure 3:**
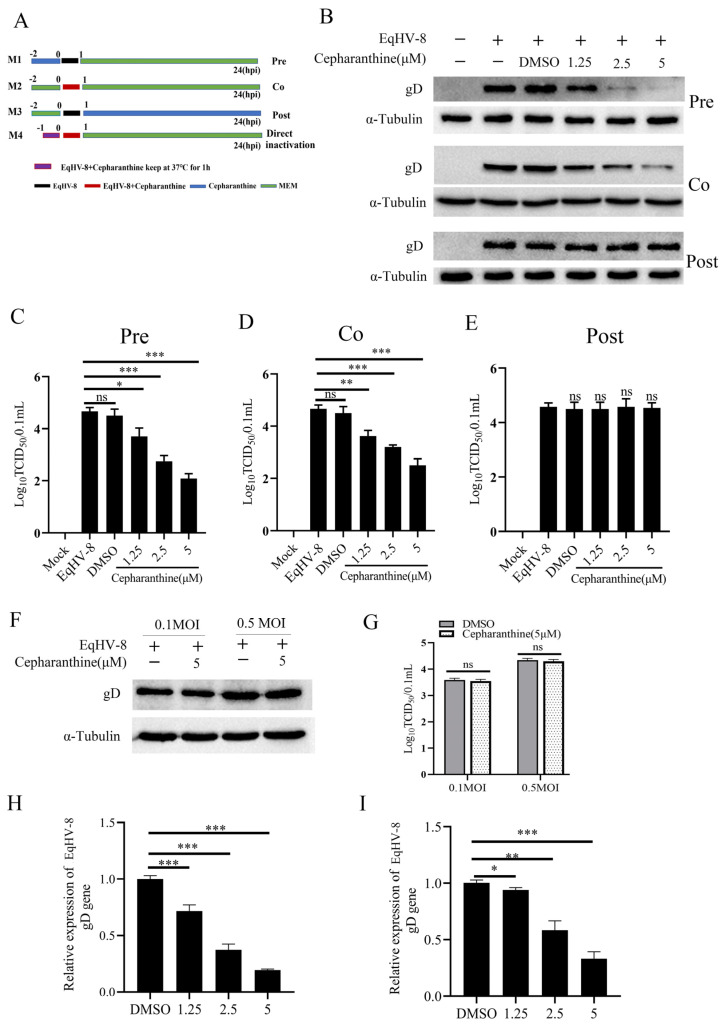
Cepharanthine exerts its primary antiviral effects against EqHV-8 during the early stages of infection. (**A**) Schematic representation of the treatment protocol. NBL-6 cells were infected with EqHV-8 SDLC66 (MOI = 0.1) and exposed to cepharanthine at different stages: prior to infection (pretreatment), during infection (co-treatment), and post-infection (post-treatment). A direct inactivation assay was also conducted, where cepharanthine was incubated with EqHV-8 to assess the immediate virucidal effects. (**B**) NBL-6 cells were treated with varying concentrations of cepharanthine (0, 1.25, 2.5, and 5 μM) during each treatment stage (pretreatment, co-treatment, and post-treatment) and infected with EqHV-8 (MOI = 0.1). At 24 hpi, gD expression was evaluated by Western blot, while progeny virus titers were measured using TCID_50_ assays (**C**–**E**). α-Tubulin served as a loading control. Data are presented as the mean ± standard deviation from three independent experiments. Significance was determined as follows: * *p* < 0.05; ** *p* < 0.01; *** *p* < 0.001; ns: not significant. (**F**–**G**) In the virucidal assay, cepharanthine (5 µM) or DMSO was incubated with EqHV-8 (MOI = 0.1 and 0.5) for 2 h at 37 °C. Following incubation, the treated virus was used to infect NBL-6 cells. gD protein levels and progeny titers were assessed at 24 hpi via Western blot and TCID_50_ assays, respectively. (**H**) For the adsorption assay, NBL-6 cells were incubated with a cepharanthine (5 µM) or DMSO-EqHV-8 mixture (MOI = 0.1) at 4 °C for 1 h. The cells were then washed and incubated in 3% FBS MEM at 37 °C for 24 h before gD expression was quantified by qPCR. (**I**) In the internalization assay, NBL-6 cells were pretreated with cepharanthine (5 µM) for 12h, followed by incubation with EqHV-8 (MOI = 0.1) at 4 °C for 1 h. After washing, cells were incubated with either cepharanthine or DMSO for an additional hour at 37 °C, washed with citrate buffer, and further incubated in 3% FBS MEM at 37 °C. gD expression was detected using qPCR (**I**) at 24 hpi. * *p* < 0.05; ** *p* < 0.01; *** *p* < 0.001; ns: not significant.

To determine which stage of the EqHV-8 life cycle is affected by cepharanthine, we conducted time-course analysis experiments with NBL-6 cells. (Figure 3A). NBL-6 cells were harvested at various stages to quantify the protein expression of gD at 24 hpi. The results, as illustrated in Figure 3B, clearly demonstrated that gD expression was significantly lower in two specific treatment groups: the cepharanthine pretreatment group (M1; Pre) and the cepharanthine and EqHV-8 co-treated group (M2; Co), as compared to the cepharanthine post-treatment group (M3; Post). This observation strongly suggests that cepharanthine primarily inhibits EqHV-8 infection at the early stages of the viral life cycle.

Simultaneously, we assessed the viral progeny’s titer at 24 hpi using the TCID_50_ assay. Notably, the results in Figure 3C,D indicate a dose-dependent reduction in viral progeny titer in the M1 (Pre) and M2 (Co) groups, further confirming the inhibitory effect of cepharanthine at early stages. However, interestingly, no significant changes in viral progeny titer were observed in the M3 (Post) group, as shown in Figure 3E.

Furthermore, we introduced M4, which represents a direct inactivation assay of cepharanthine against EqHV-8. In this assay, a mixture of cepharanthine and EqHV-8 (at MOI values of 0.1 or 0.5) was incubated for 1 h, and then the virus particles were used to infect NBL-6 cells. The cells and their respective supernatants were collected to assess EqHV-8 infection via Western blot and TCID_50_ assays. Surprisingly, the results shown in Figure 3F,G revealed no significant difference in progeny virus titers and gD protein expression between the cepharanthine-treated groups and the DMSO-treated control groups. This compelling evidence leads to the conclusion that cepharanthine does not have a direct virucidal effect against EqHV-8.

Finally, to investigate whether the antiviral activity of cepharanthine is mediated through its impact on viral adsorption or internalization within NBL-6 cells, we conducted viral adhesion and virus entry assays. Our data in Figure 3H,I clearly demonstrate that at both the adsorption and internalization stages, NBL-6 cells pre-treated with cepharanthine showed significantly lower gD protein expression compared to those pre-treated with DMSO. These findings strongly support the idea that cepharanthine exerts its antiviral effects by preventing EqHV-8 infection at the adsorption and internalization stages of the viral life cycle.

### 3.4. Cepharanthine Alleviates EqHV-8-Induced Oxidative Stress via HO-1 Activation 

**Figure 4:**
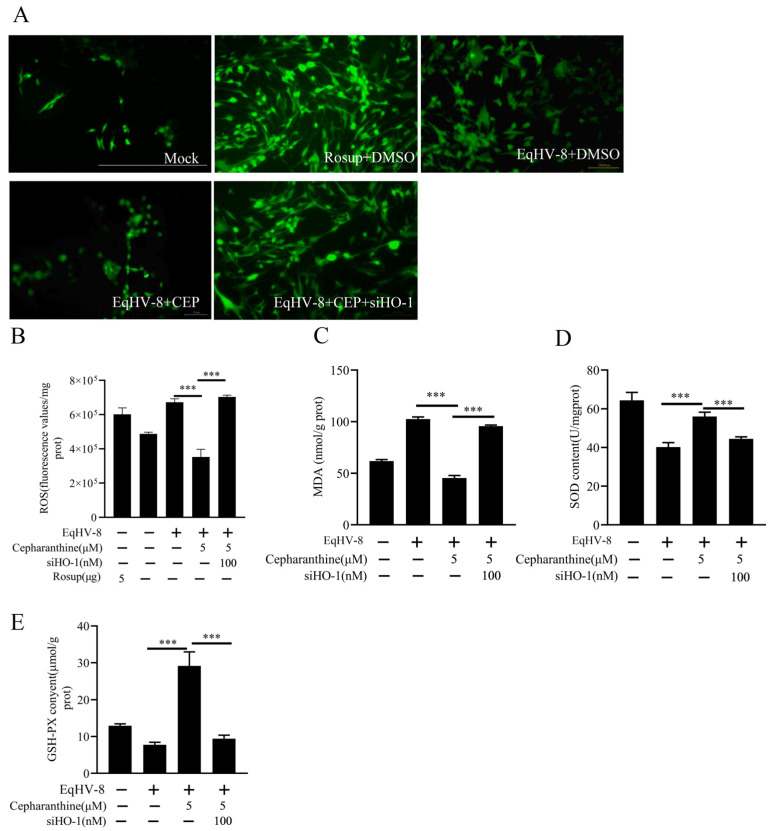
Cepharanthine attenuates oxidative stress triggered by EqHV-8 infection through HO-1 activation. NBL-6 cells were subjected to siHO-1 transfection for 10 h, followed by a 2 h pre-treatment with cepharanthine (5 µM). Subsequently, the cells were infected with EqHV-8 SDLC66 (MOI = 0.1) for 1 h. ROS production was evaluated using the DCFH-DA assay, with images captured by a Leica DMi 8 fluorescence microscope (**A**), where green fluorescence indicates ROS generation. Scale bar: 100 µm. Additionally, the fluorescence intensity was quantified using a Tecan Spark microplate reader (**B**). The impact of cepharanthine on MDA (**C**), SOD (**D**), and GSH-PX (**E**) levels was assessed via the Multiskan Spectrum. Statistical significance was denoted by *** *p* < 0.001.

Recent evidence has demonstrated that cepharanthine can reduce oxidative stress in mammalian cells by activating the HO-1-mediated antioxidant system [17]. Therefore, our study aimed to explore the relationship between the anti-EqHV-8 activity of cepharanthine and its antioxidant properties. To address this question, we measured the levels of ROS in NBL-6 cells after pretreatment with 5 μM cepharanthine and subsequent EqHV-8 infection. Interestingly, EqHV-8 infection led to a significant increase in ROS levels (observed in the PBS group). However, treatment with cepharanthine was able to reduce ROS levels and MDA levels in EqHV-8 infected cells, as shown in Figure 4A–C. Additionally, cepharanthine administration also increased the expression of other important antioxidant biomarkers, namely SOD (Figure 4D) and GSH (Figure 4E). Importantly, these effects were reversible when specific siRNAs targeting HO-1 were used. These findings suggest that cepharanthine has the potential to restore the redox balance in NBL-6 cells by activating HO-1. 

### 3.5. Cepharanthine Exerts Anti-EqHV-8 Activity by Upregulating Heme Oxygenase-1 (HO-1)/Biliverdin (BV) Expression

**Figure 5:**
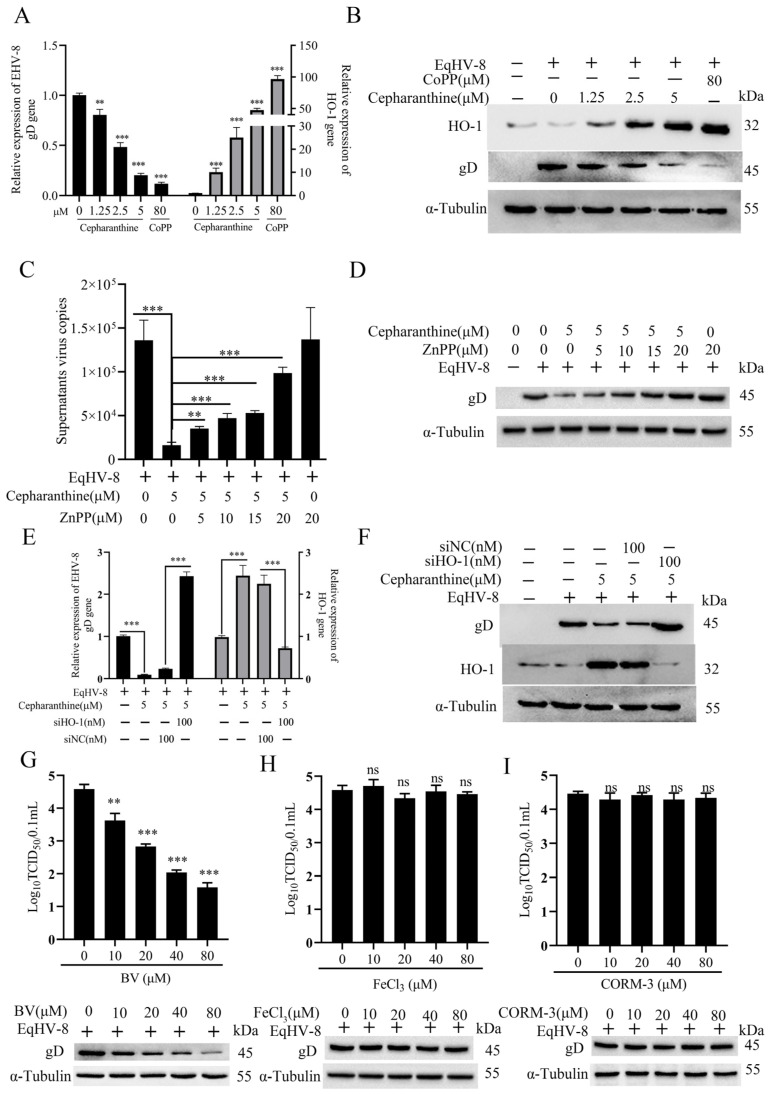
Cepharanthine enhances ho-1/bv expression to suppress EqHV-8 replication. NBL-6 cells were treated with varying concentrations of cepharanthine (0, 1.25, 2.5, and 5 μM) or CoPP (80 μM) for 2 h before being infected with EqHV-8 SDLC66 at an MOI of 0.1. Cells were harvested at 24 hpi to analyze the expression levels of HO-1 and gD via qPCR (**A**) and Western blot (**B**). Statistical significance was denoted as ** *p* < 0.01, *** *p* < 0.001. In a separate experiment, NBL-6 cells were pre-treated with cepharanthine (5 μM) and various concentrations of ZnPP (5, 10, 15, and 20 μM) for 2 h, followed by infection with EqHV-8 SDLC66 at an MOI of 0.1. Cells were then collected at 24 hpi for HO-1 and gD expression analysis using qPCR (**C**) and Western blot (**D**). ** *p* < 0.01; *** *p* < 0.001. In another experiment, NBL-6 cells were transfected with either siHO-1 or siNC for 12 h and subsequently infected with EqHV-8 SDLC66 (MOI = 0.1) in the presence or absence of cepharanthine (5 μM) for 24 h. The expression of HO-1 and gD was assessed using qPCR (**E**) and Western blot (**F**), with GAPDH serving as the internal control. *** *p* < 0.001. NBL-6 cells were also pre-treated with different concentrations of Biliverdin (10, 20, 40, and 80 μM) and infected with EqHV-8 SDLC66 (MOI = 0.1). The expression of gD at the protein level and progeny virus titers were determined using Western blot and TCID_50_ assays (**G**). Additionally, similar experiments were conducted in NBL-6 cells at 24 hpi using CORM-3 and FeCl_3_ at specified concentrations (**H**,**I**). GAPDH was used as a reference gene, while α-Tubulin was the loading control. Data are presented as the mean ± standard deviation from three independent experiments, with ** *p* < 0.01, *** *p* < 0.001, and ns indicating non-significant differences.

Cepharanthine has been previously documented in the literature for its remarkable pharmacological properties, including its well-established roles as an antioxidant, anti-inflammatory agent, and antiviral agent, as indicated by references [15,16]. In the context of cellular stress response and cytoprotection, heme oxygenase-1 (HO-1) emerges as a pivotal cytoprotective protein, instrumental in orchestrating immediate protective responses against various cellular insults, particularly oxidative stress, in mammalian cells [23]. To investigate the relationship between the anti-EqHV-8 activity of cepharanthine and the modulation of HO-1 expression, the NBL-6 cells were pre-treated with different concentrations of cepharanthine for 2 h. After that, they were infected with EqHV-8 SDLC66. The levels of HO-1 and gD expression were evaluated using Western blot and qPCR at 24 hpi. We used CoPP (an HO-1 inducer) as a positive control. Figure 5A,B demonstrate that cepharanthine increases HO-1 expression at both the protein and mRNA levels compared to the group treated with DMSO (0 μM cepharanthine). To further confirm the relationship between cepharanthine-induced HO-1 activation and its activity against EqHV-8, NBL-6 cells were co-treated with cepharanthine (5 μM) and various concentrations of ZnPP (a known HO-1 inhibitor) before being infected with EqHV-8 SDLC66 at 0.1 MOI. The interplay between HO-1 expression and EqHV-8 replication was carefully assessed using Western blot and qPCR at 24 hpi. As shown in Figure 5C,D, our results indicate that cepharanthine significantly upregulates HO-1 expression while simultaneously reducing gD expression in NBL-6 cells. Interestingly, the administration of ZnPP mitigated the anti-EqHV-8 activity of cepharanthine. The cytotoxicity of CoPP and ZnPP was tested and is shown in Supplementary Figure S1. Furthermore, we explored the impact of small interfering RNA targeting HO-1 (siHO-1) on cepharanthine-mediated anti-EqHV-8 effects in NBL-6 cells. Following transfection with siHO-1, treatment with cepharanthine (5 μM), and EqHV-8 SDLC66 (MOI = 0.1) infection, both HO-1 expression and gD expression were scrutinized through Western blot and qPCR assays. Notably, our data, as depicted in Figure 5E,F, unveil that siHO-1 can effectively counteract the anti-EqHV-8 effects mediated by cepharanthine. In summation, these findings revealed that the efficacy of cepharanthine against EqHV-8 is intricately dependent on the activation of HO-1.

Previous findings have reported that HO-1 helps fight viruses by working closely with its byproducts like BV, carbon monoxide (CO), and iron [24,25,26,27,28]. To gain a deeper understanding of which of these metabolites may exert suppressive effects on EqHV-8 replication, we first test the concentrations of BV, carbon monoxide (CO), and iron using kits. As Supplementary Figure S2A–C shows, cepharanthine increases BV, CO, and iron generation in a dose-dependent manner. Further, we pre-treated NBL-6 cells with varying concentrations of BV, CORM-3 (a CO-releasing molecule), or FeCl_3_ for a duration of 1 h prior to EqHV-8 SDLC66 (MOI = 0.1) infection. The concentrations of these molecules were thoughtfully selected based on preliminary cytotoxicity assessments (Supplementary Figure S2D). Subsequently, we meticulously examined gD expression levels and progeny virus production through Western blot and TCID_50_ assays. Notably, we documented that BV treatment leads to a significant reduction in both gD expression and progeny virus titers in NBL-6 cells, with a clear dose-dependent effect (Figure 5G). In contrast, no significant alterations in gD expression or progeny virus titers were distinguished in the FeCl_3_ or CORM-3 treated groups when compared to the control group (Figure 5H–I). In light of these results, our research firmly suggests that cepharanthine’s anti-EqHV-8 effects are fundamentally mediated through the upregulation of HO-1 and its downstream metabolite, BV.

### 3.6. Cepharanthine Inhibits EqHV-8 Replication by Activating the AMPK and Nrf2/HO-1 Signaling Pathways

It has been well documented that AMPK plays a pivotal role in the regulation of cellular energy homeostasis, oxidative stress, inflammation, and autophagy in mammalian cells, as elucidated in previous studies [29]. Simultaneously, recent investigations have underscored the significance of the Nrf2/HO-1 signaling pathway as a key regulator of oxidative stress, inflammation, and apoptosis [30,31,32]. In light of these established findings, our study embarked upon an exploration of the impact of cepharanthine on the AMPK and Nrf2/HO-1 signaling pathways within NBL-6 cells.

**Figure 6:**
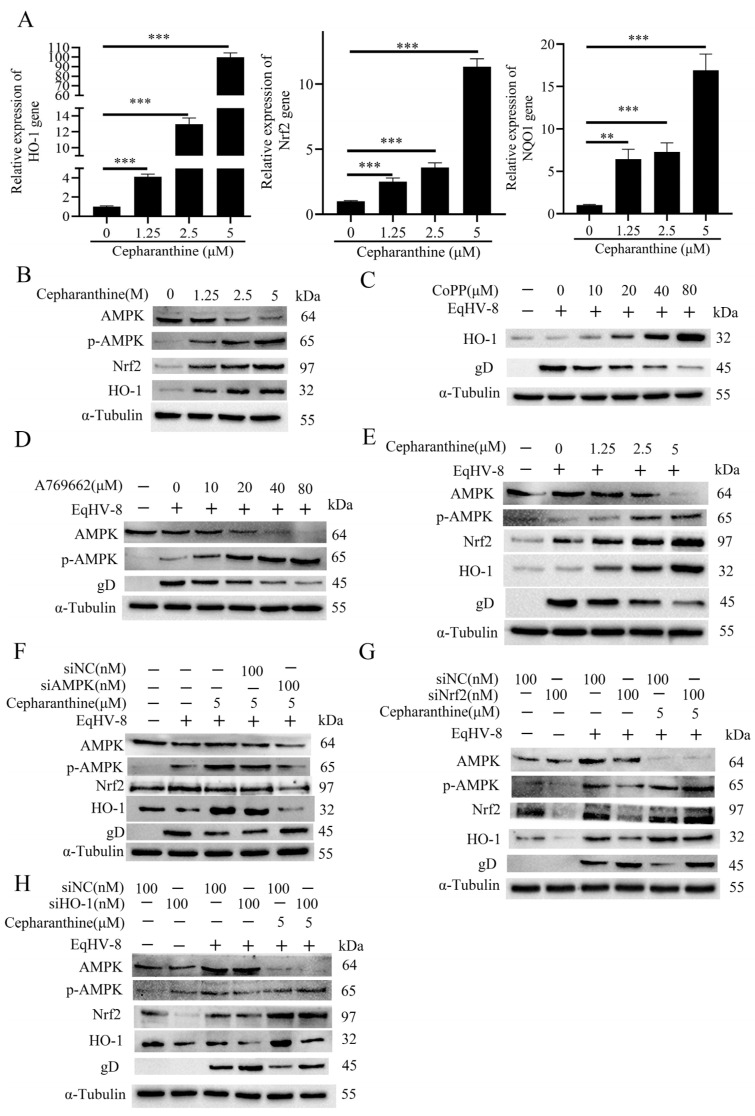
Cepharanthine’s effects on the AMPK and Nrf2/HO-1 signaling pathways. (**A**) NBL-6 cells were treated with cepharanthine in essential medium for 24 h at 37 °C. The mRNA expression levels of HO-1, Nrf2, and NQO1 were measured by qPCR. Data are presented as mean ± standard deviation (error bars) from at least three independent experiments. Statistical significance was indicated by ** *p* < 0.01 and *** *p* < 0.001. (**B**) Protein levels of AMPK, p-AMPK, Nrf2, and HO-1 were examined in the treated cells using Western blot analysis. (**C**) NBL-6 cells were pretreated with different concentrations of CoPP (10, 20, 40, and 80 µM) at 37 °C for 2 h, followed by infection with EqHV-8 SDLC66 (MOI = 0.1). Post-infection, the cells were incubated with fresh media containing the same concentrations of CoPP, and HO-1 and glycoprotein D (gD) expression was analyzed by Western blot. (**D**) NBL-6 cells were pretreated with varying concentrations of A769662, an AMPK activator (10, 20, 40, and 80 µM), for 2 h at 37 °C. Afterward, cells were infected with EqHV-8 SDLC66 (MOI = 0.1) for 1 h, and the medium was replaced with 3% FBS MEM containing A769662 at the respective doses. Protein levels of AMPK, p-AMPK, and gD were assessed by Western blot. (**E**) NBL-6 cells were pretreated with cepharanthine at concentrations of 0, 1.25, 2.5, or 5 µM for 2 h at 37 °C, followed by infection with EqHV-8 SDLC66 (MOI = 0.1) for 1 h. The medium was then replaced with 3% FBS MEM containing cepharanthine at the corresponding concentrations, and the cells were harvested to analyze the expression of AMPK, p-AMPK, Nrf2, HO-1, and gD using Western blot. (**F**–**H**) NBL-6 cells were individually transfected with siAMPK (**F**), siNrf2 (**G**), siHO-1 (**H**), or siNC for 10 h, followed by infection with EqHV-8 SDLC66 (MOI = 0.1). At 24 hpi, the expression levels of AMPK, p-AMPK, Nrf2, HO-1, and gD were determined using Western blot analysis. GAPDH served as the internal reference, and α-Tubulin was used as the loading control.

Initially, NBL-6 cells underwent incubation with varying concentrations of cepharanthine at 37 °C for 24 h, with CoPP employed as a positive control. Subsequently, the cellular expression levels of HO-1, Nrf2, and NQO1 were assessed through qPCR. The results unveiled a noteworthy upregulation of HO-1, Nrf2, and NQO1 following cepharanthine treatment in NBL-6 cells (Figure 6A). Concurrently, Western blot analysis revealed a dose-dependent increase in the protein levels of HO-1, Nrf2, and phosphorylated AMPK (p-AMPK) following cepharanthine treatment (Figure 6B). These observations suggest the ability of cepharanthine to activate AMPK and the Nrf2/HO-1 signaling pathway. Subsequently, our investigation delved deeper into ascertaining whether the activation of the AMPK and Nrf2/HO-1 signaling pathways is indeed linked to the anti-EqHV-8 (Equid herpesvirus 8) activity exhibited by cepharanthine. To elucidate this connection, NBL-6 cells were subjected to treatment with varying doses of CoPP (an HO-1 inducer) or A769662 (an AMPK activator), as determined through preliminary cytotoxicity assays (as provided in Supplementary Figure S3). These cells were subsequently infected with EqHV-8 SDLC66. As illustrated in Figure 6C, CoPP treatment resulted in the augmentation of endogenous HO-1 expression, coupled with a concomitant reduction in gD expression in NBL-6 cells, exhibiting a dose-dependent pattern. Similarly, the A769662 treatment yielded comparable outcomes, characterized by a significant downregulation of gD and AMPK alongside a conspicuous upregulation of p-AMPK (Figure 6D). Next, we proceeded to elucidate the influence of cepharanthine on the AMPK and Nrf2/HO-1 signaling pathways within EqHV-8-infected NBL-6 cells. Our data unequivocally demonstrated that cepharanthine induced a noticeable increase in the expression levels of HO-1, Nrf2, and p-AMPK while concurrently reducing the expression of EqHV-8 gD in NBL-6 cells (Figure 6E).

To further corroborate the role of AMPK, Nrf2, and HO-1 activation in modulating EqHV-8 replication, we employed small interfering RNAs (siRNAs) targeting these specific factors (siAMPK, siNrf2, and siHO-1). The siRNA availability of siAMPK, siNrf2, and siHO-1 were screened and shown in Supplementary Figure S4. Next, the NBL-6 cells were transfected with siAMPK, siNrf2, siHO-1, or siNC (negative control) for 12 h and subsequently infected with EqHV-8 SDLC66. Following infection, the cellular expression levels of AMPK, p-AMPK, Nrf2, and HO-1 were assessed via Western blot assays, and EqHV-8 replication was evaluated. As illustrated in Figure 6F, siAMPK-treated NBL-6 cells exhibited a reduction in both AMPK and gD expression compared to siNC-treated cells. Similarly, siNrf2-treated NBL-6 cells displayed a conspicuous decrease in Nrf2 expression and a notable increase in gD expression (Figure 6G), while siHO-1-treated NBL-6 cells exhibited a marked reduction in HO-1 expression and a concurrent increase in gD expression (Figure 6H).

### 3.7. Cepharanthine Activates AMPK by Increasing the AMP/ADP-to-ATP Ratio

**Figure 7:**
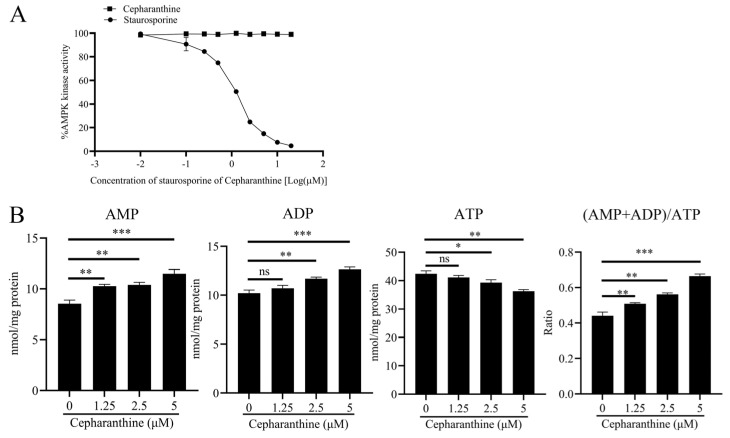
Cepharanthine activates AMPK by modulating the ADP/AMP-to-ATP ratio rather than directly binding to AMPK. (**A**) Cepharanthine or staurosporine was diluted in 10% DMSO, and 5 μL of this dilution was added to a 50 μL reaction mixture. The AMPK inhibition assay was performed at 30 °C for 40 min using the kinase-glo plus luminescence kinase assay kit. (**B**) NBL-6 cells were treated with cepharanthine at various concentrations (0, 1.25, 2.5, or 5 µM) for 24 h, after which the cells were harvested to assess AMP, ADP, ATP levels, and the AMP/ADP-to-ATP ratio. Data are presented as the mean ± standard deviation (error bars), normalized from at least three independent experiments. Statistical significance is indicated as follows: * *p* < 0.05; ** *p* < 0.01; *** *p* < 0.001; ns: not significant.

AMPK is a well-established sensor of the AMP/ATP ratio and comprises catalytic α subunits in addition to regulatory β and γ subunits [33]. Notably, AMPK can be activated both physiologically and allosterically. Physiological activation of AMPK involves the phosphorylation of Thr-172 within the activation loop of the N-terminal kinase domain (KD) in its α-catalytic subunit. Extensive literature has documented the involvement of liver kinase B1 (LKB1) and calcium/calmodulin-dependent protein kinase 2 (CAMKK2) in phosphorylating Thr-172 of the AMPK α subunit [34,35]. In contrast, allosteric activation of AMPK occurs through the direct binding of AMP or its analogs to the β subunit of AMPK. It is important to note that the effects of AMP and ADP are counteracted by ATP, resulting in AMPK regulation that is contingent on the AMP/ADP-to-ATP ratio [36]. In the context of this study, our investigation delves into elucidating the mechanism(s) responsible for cepharanthine-mediated AMPK activation. Initially, we assessed the impact of cepharanthine on AMPK activity utilizing a kinase-Glo Plus luminescence kinase assay kit. As mentioned in Figure 7A, the positive control Staurosporine (an AMPK inhibitor) effectively suppressed AMPK activity in a dose-dependent manner (with a 50% inhibitory concentration [IC50] of 1.1 µM). However, we observed that the activity of AMPK did not exhibit significant alterations following treatment with cepharanthine. Subsequently, we sought to examine the effects of cepharanthine treatment on the levels of AMP, ADP, and ATP within NBL-6 cells. Our findings revealed that cepharanthine induced a dose-dependent increase in cellular AMP and ADP levels, coupled with a concomitant decrease in ATP levels (at concentrations of 1.25, 2.5, and 5 μM). Particularly noteworthy was the effect of 5 μM cepharanthine treatment, which resulted in a 1.5-fold increase in the ADP/AMP-to-ATP ratio within NBL-6 cells (Figure 7B). These observations collectively suggest that cepharanthine primarily activates AMPK by elevating the ADP/AMP-to-ATP ratio rather than through direct binding mechanisms.

### 3.8. Cepharanthine Suppresses EqHV-8 Infection In Vivo

**Figure 8:**
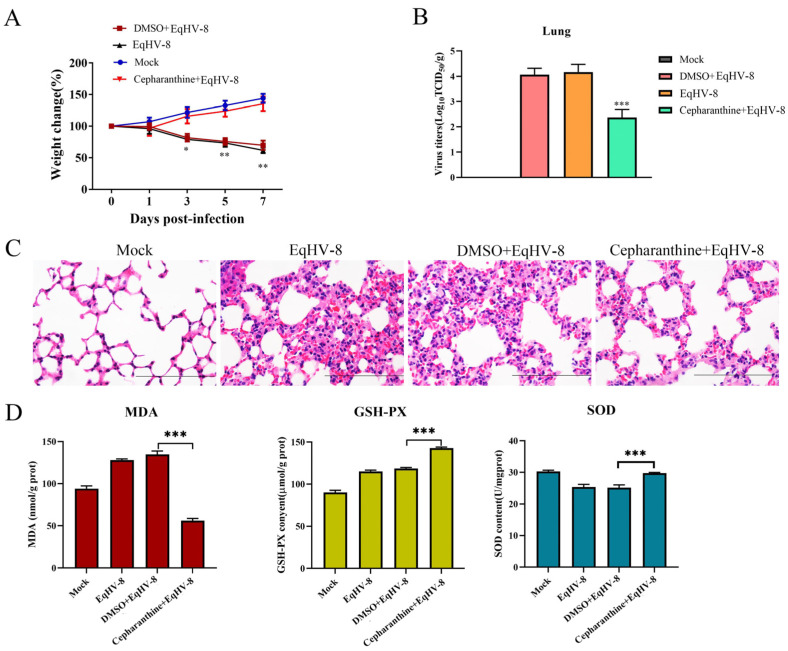
Cepharanthine shows antiviral activity against EqHV-8 in vivo. Mice (n = 5/group) received intraperitoneal injections of DMSO and cepharanthine before infection with EqHV-8. The DMEM treatment served as the mock treatment group. (**A**) Body weight changes in each group were measured at indicated time points. (**B**) Lung samples were collected at 7 dpi, and EqHV-8 titers in lung tissue were determined via TCID_50_ assays. (**C**) The lung tissues of mice in the four groups were fixed at 7 dpi and sectioned for hematoxylin and eosin (H&E) staining. Scale bar, 100 µm. (**D**) MDA, SOD, and GSH-PX activity was examined in serum samples from the mice at 7 dpi. All data are expressed as the mean ± S.D. * *p* < 0.05; ** *p* < 0.01; *** *p* < 0.001.

Previous research has established that cepharanthine demonstrates substantial antiviral efficacy in vitro. However, its in vivo antiviral effect remains relatively unexplored. This study aimed to investigate the antiviral potential of cepharanthine in BALB/c mice, as shown in Table 3. In this study, mice infected with EqHV-8 experienced weight loss, while those treated with cepharanthine gained weight significantly (Figure 8A). The replication of EqHV-8 in lung tissues was measured using TCID_50_ assays (Figure 8B), which revealed significantly lower levels of EqHV-8 replication in the cepharanthine-treated group compared to the DMSO-treated EqHV-8 group. These results align with reduced lung damage caused by EqHV-8 in the cepharanthine group, as shown by mild thickening of alveolar walls and infiltration of inflammatory cells (Figure 8C). Additionally, we assessed the levels of oxidative stress markers in the serum of both groups of mice. Notably, the cepharanthine-treated group had significantly lower levels of MDA in the serum compared to the DMSO-treated EqHV-8 group (Figure 8D). In contrast, the expression levels of SOD and GSH-PX were significantly higher in the cepharanthine group. These findings suggest that cepharanthine may inhibit viral replication by reducing oxidative stress in mice.

## 4. Discussion

In recent years, there has been a noticeable increase in cases of rhinopneumonitis and miscarriage within the context of large-scale donkey farming operations in China. These occurrences have resulted in significant economic losses and have posed challenges to the development of the donkey farming industry [37,38]. Previous research conducted by our group has established that EqHV-8 is responsible for causing rhinopneumonitis, miscarriage, and neurological disorders in donkeys [11,12,13]. Furthermore, an epidemiological investigation revealed a high prevalence of EqHV-8 infections among donkeys in Shandong Province, China, at 38.7% (457 out of 1180) [39]. These findings highlight EqHV-8 as the most critical pathogen affecting donkeys in Chinese farming environments. Since there are currently no effective anti-EqHV-8 therapeutics or vaccines, the urgent need to develop antiviral drugs to combat EqHV-8 has emerged.

In the recent scientific literature, cepharanthine has gained recognition for its proven antiviral activity against various pathogens, including SARS-CoV-2, HSV-1, and the dengue virus (DENV) [15,18,40]. The aim of this study was to investigate the antiviral and antioxidant properties of cepharanthine in cells susceptible to EqHV-8. Our research revealed that cepharanthine significantly reduced the expression of viral gD protein and inhibited the production of progeny virus in infected RK-13 and NBL-6 cells, as shown in Figure 1 and Figure 2. Additionally, cepharanthine exhibited inhibitory effects against EqHV-8 infection in a murine model (Figure 8). To understand the underlying mechanisms, we conducted a detailed analysis of cepharanthine’s impact on EqHV-8 infection in vitro. Our findings demonstrated that cepharanthine primarily exerts its inhibitory effects during the initial stages of viral binding and internalization without possessing virucidal properties (Figure 3). These results are consistent with previous studies that reported cepharanthine’s inhibitory effects against DENV infection [41].

Oxidative stress is a common occurrence in cells infected with a virus. It is characterized by a disruption in antioxidant defenses, which leads to damage in the cells and tissues [42]. Numerous studies have shown a relationship between viral infections and oxidative stress. For example, the hepatitis C virus induces oxidative stress in liver tissue [43], and SARS-CoV-2 infection triggers the release of neutrophil-induced ROS, worsening tissue damage [44]. The influenza virus also promotes the formation of reactive peroxynitrite and causes oxidative stress in pulmonary tissue [45,46]. Additionally, the porcine reproductive and respiratory syndrome virus (PRRSV) increases the production of ROS and MDA while decreasing the generation of SOD and GSH in susceptible cells and lung tissues of pigs [42]. Our study demonstrates that treatment with cepharanthine effectively reduces oxidative stress damage caused by EqHV-8 in NBL-6 cells. This is achieved by suppressing ROS production and MDA levels, highlighting its potential as an antioxidant agent in the context of viral infections, as depicted in Figure 4.

HO-1 is an important enzyme known for its cytoprotective properties. It has been shown to have anti-inflammatory, antiviral, and antioxidant properties [47]. Previous studies have demonstrated that HO-1 and its metabolites, including BV, iron ions, and CO, can effectively inhibit various viruses, such as human immunodeficiency virus type 1, PRRSV, pseudorabies virus, hepatitis A virus, and porcine circovirus 3 [48,49,50,51,52]. Our research has revealed that cepharanthine can inhibit EqHV-8 by increasing the expression of HO-1 in a dose-dependent manner. Interestingly, it was the metabolite BV of HO-1, rather than iron or CO, that exhibited antiviral effects against EqHV-8 (Figure 5).

Nrf2 plays a crucial role in regulating cellular redox balance and the antioxidant response in mammals [53,54]. The Nrf2/HO-1 signaling pathway is key in responding to oxidative stress and viral pathogenesis [31]. In our study, we discovered that cepharanthine inhibits EqHV-8 infection by activating the Nrf2/HO-1 signaling pathway. Treatment with cepharanthine increased the expression of Nrf2 and HO-1 while reducing gD expression in NBL-6 cells. These effects were reversed when small interfering RNAs (siRNAs) targeting Nrf2 and HO-1 were transfected, confirming the necessity of activating the Nrf2/HO-1 pathway for the anti-EqHV-8 effect of cepharanthine (Figure 6).

AMPK is a heterotrimeric protein complex that serves as the cell’s primary energy sensor, essential for maintaining energy balance [55,56]. The AMPK signaling pathway is mainly activated when the ratio of AMP/ATP or ADP/ATP increases [57]. Recent studies have also linked AMPK to the regulation of antiviral defense and response to oxidative stress [58,59,60]. Our study showed that in NBL-6 cells, cepharanthine treatment increased p-AMPK protein expression in a dose-dependent manner. Furthermore, treating with siRNAs that target AMPK reverses the anti-EqHV-8 effects of cepharanthine, as depicted in Figure 6. This supports the idea that cepharanthine exerts its anti-EqHV-8 effect by activating the AMPK signaling pathway.

**Figure 9:**
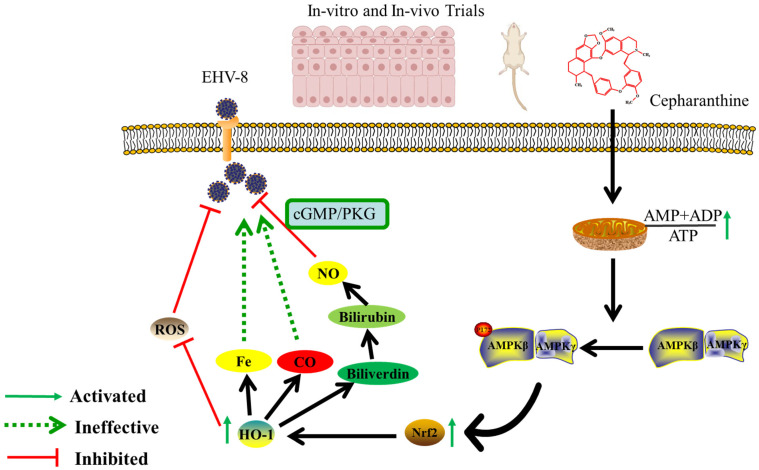
Cepharanthine activates AMPK by increasing the AMP/ADP-to-ATP ratio, which activates Nrf2 and its downstream factor, HO-1. Biliverdin (BV), a metabolic product of HO-1, directly or indirectly mediates the anti-EqHV-8 effect of cepharanthine via cGMP/PKG signaling. Cepharanthine also decreases oxidative stress by suppressing ROS and MDA levels and increasing GSH and SOD activity, thereby promoting the inhibition of EqHV-8 replication.

AMPK is composed of three subunits: α, β, and γ. The α subunit consists of a protein kinase domain (KD) at the N-terminal, which is connected to a regulatory domain at the C-terminal. The β subunit contains a carbohydrate-binding module (CBM) or glycogen-binding domain (GDB), and its C-terminal region acts as a scaffold, facilitating interactions with the α and γ subunits of AMPK. The γ subunit consists of four cystathionine-β-synthase (CBS) domains, which are essential for nucleotide binding [29]. The activation of AMPK in the body involves the phosphorylation of Thr-172 within the loop of the α catalytic subunit. This process is regulated by LKB1 or CaMKK2 as part of the upstream signaling cascade [34,61]. Previous studies have demonstrated that AMP binding to the γ subunit of AMPK significantly enhances LKB1-dependent phosphorylation at Thr172 [62]. Certain indirect AMPK activators, such as metformin and berberine, promote the accumulation of AMP or calcium, leading to the physiological activation of AMPK. On the other hand, allosteric AMPK activation occurs when AMP or compounds that mimic it directly interact with specific subunits of AMPK, causing changes in the conformation of the AMPK complex, even in the absence of changes in ADP, ATP, or AMP levels. Direct AMPK activators, such as A-769662 and salicylate, induce allosteric AMPK activation by binding to specific domains of AMPK [63]. In our study, we found that treatment with cepharanthine did not directly affect AMPK phosphorylation by binding to the α subunit of AMPK (Figure 7A). However, we did observe that cepharanthine treatment increased AMP and ADP levels while decreasing ATP levels in a dose-dependent manner in NBL-6 cells, thereby promoting AMPK activation (Figure 7B). Consistent with our findings, a previous study reported that the antimalarial drug artesunate decreases ATP levels in the host cell by disrupting the replication process of the porcine reproductive and respiratory syndrome virus (PRRSV) and interfering with the host cell’s metabolism [60]. In our study, we have discovered that cepharanthine has the potential to prevent EqHV-8 infection by decreasing ATP levels in the host cell, thus disrupting the viral replication process. Overall, our study provides a comprehensive understanding of the potential of cepharanthine as an antiviral and antioxidant agent against EqHV-8 infection. Its multifaceted mechanisms of action include inhibiting viral binding and internalization, suppressing EqHV-8-induced oxidative stress, inducing HO-1 expression, and activating the Nrf2/HO-1 and AMPK signaling pathways (Figure 9). Furthermore, our findings have demonstrated the powerful anti-EqHV-8 effect of cepharanthine both in vitro and in vivo. However, there are still limitations to the present study and further investigation is required to determine the impact of cepharanthine on respiratory function in animal experiments, as well as to explore its direct targets.

## 5. Conclusions

Altogether, our findings demonstrate that cepharanthine significantly inhibits EqHV-8 infection both in vitro and in vivo. Furthermore, cepharanthine exerts these inhibitory effects via AMPK by augmenting the AMP/ADP-to-ATP ratio, thereby further activating the Nrf2/HO-1 signaling pathway. Additionally, the downstream metabolite of HO-1 and BV can also directly or indirectly mediate cepharanthine’s anti-EqHV-8 effect through the cGMP/PKG signaling pathway. Notably, cepharanthine also mitigates the oxidative stress injury induced by EqHV-8 infection by enhancing HO-1 expression. The findings presented here collectively suggest that cepharanthine has therapeutic potential against EqHV-8 infections. However, further research is required to assess its efficacy and safety in horses and donkeys prior to its consideration for clinical application within equine populations.

## Figures and Tables

**Table 1:** List of primers used in this study.

Genes	Forward Primer (5′-3′)	Reverse Primer (5′-3′)
ORF72	CCCACGTGTGCAACGCCTAT	ATACAGTCCCGAGGCAGAGT
HO-1	AGTTCATGAAGAACTTTCAGAA	TACCAGAAGGCCATGTCC
Nrf2	ATTCAATGATTCTGACTCTG	CGTATCCCCAGAAGAATGTA
NQO1	CATGTACTCTCTGCAAGGGA	TCCCAAATATTCTCCAGGCG
GAPDH	GTCTCCTCTGACTTCAACAGCG	ACCACCCTGTTGCTGTAGCCAA

**Table 2:** siRNA sequences utilized in this study.

Primers	Sequences (5′-3′)
siHO-1-①	GGTCCTCACACTCAGCTTT
siHO-1-②	CCACCAAGTTCAAGCAGCT
siHO-1-③	CCACCAAGTTCAAGCAGCT
siNrf2-①	CTCCTTAAGAAGCAACTCA
siNrf2-②	GTCACTCTCTGAACTTCTA
siNrf2-③	GACATTCCCATTTGTAGAT
siAMPK-①	CCTTTCTGGTGTGGATTAT
siAMPK-②	GGAGAGCTATTTGATTTATA
siAMPK-③	GGGATCCGTTAGCAACTAT

**Table 3:** Treatment groups in cepharanthine efficacy study against EqHV-8 inoculation.

Numbering	Group	Inoculation
1	Mock	100 μL MEM
2	Virus	100 μL EqHV-8
3	DMSO	100 μL DMSO + 100 μL EqHV-8
4	Cepharanthine	Cepharanthine + 100 μL EqHV-8

## Data Availability

All data generated or analyzed during this study are available from the corresponding author upon reasonable request.

## Supplementary Materials

The following supporting information can be downloaded at: https://www.mdpi.com/article/10.3390/v16111765/s1, Figure S1: The cytotoxicity of CoPP, and ZnPP in NBL-6; Figure S2: The BV, CO, and iron concentrations detected by cepharanthine treatment, and the cytotoxicity of Biliverdin, FeCl3, and CORM-3 in NBL-6; Figure S3: The A769662 cytotoxicity detection in NBL-6; Figure S4: The effective siRNAs screening of AMPK, Nrf2 or HO-1 in NBL-6.

## Abbreviations

ADP	adenosine diphosphate
AHV-1	asinine herpesvirus 1
AMPK	AMP-activated protein kinase
AMP	adenosine monophosphate
Akt	protein kinase B
ATP	adenosine triphosphate
BV	biliverdin
CAMKK2	calcium/calmodulin-dependent protein kinase 2
CBM	carbohydrate-binding module
CBS	cystathionine-β-synthase
CCK-8	Cell Counting Kit-8
CO	carbon monoxide
CoPP	cobalt protoporphyrin
DAPI	4′,6-diamidino-2-phenylindole
DCF	dichlorofluorescein
DENV	dengue virus
DMSO	dimethyl sulfoxide
DTT	dithiothreitol
ECL	electrogenerated chemiluminescence
EHVs	equine herpesviruses
ELISA	enzyme-linked immunosorbent assay
EqHV-8	equid herpesvirus type 8
FBS	fetal bovine serum
gD	glycoprotein D
GAPDH	glyceraldehyde-3-phosphate dehydrogenase
GDB	glycogen-binding domain
GSK-3β	glycogen synthase kinase-3beta
GSH	glutathione
HE	hematoxylin and eosin
HIF-1	hypoxia-inducible factor 1
HO-1	heme oxygenase-1
Hpi	hours post-infection
HRP	horseradish peroxidase
HSV-1	herpes simplex virus type 1
LKB1	liver kinase B1
LPS	lipopolysaccharide
MAPK	mitogen-activated protein kinase
MDA	malonaldehyde
MEM	Modified Eagle Medium
MOI	multiplicity of infection
NBL-6	E. Derm cells
Nrf2	NF-E2-related factor 2
NQO1	NAD(P)H:quinine oxidoreductase 1
OD	optical density
PBST	phosphate-buffered saline with Tween 20
PI3K	phosphatidylinositol-3 kinase
PRRSV	porcine reproductive and respiratory syndrome virus
PVDF	polyvinylidene difluoride
qPCR	quantitative polymerase chain reaction
RK-13	rabbit kidney cells
ROS	reactive oxygen species
SARS-CoV-2	severe acute respiratory syndrome coronavirus 2
SDS-PAGE	sodium dodecyl sulfate-polyacrylamide gel electrophoresis
SOD	superoxide dismutase
SPF	specific pathogen free
TCID_50_	50% tissue culture infectious dose
VEGF	vascular endothelial-derived growth factor
ZnPP	zinc protoporphyrin
