# Supplementary material for: Protective Role of Cepharanthine Against Equid Herpesvirus Type 8 Through AMPK and Nrf2/HO-1 Pathway Activation
Source: Viruses. 2024 Nov 12;16(11):1765. doi: 10.3390/v16111765 (PMC11598968; doi:10.3390/v16111765)
Supplement: Supplementary file 1 [file viruses-16-01765-s001.zip › viruses-3273410-supplementary.pdf]

Supplementary figures and figure legends

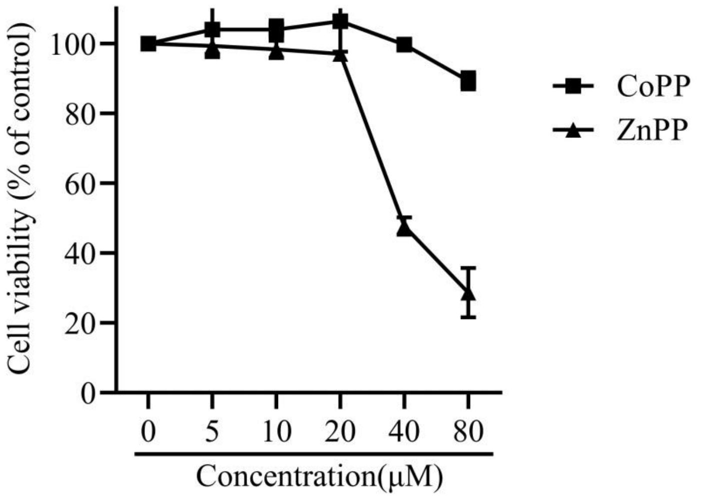

Figure S1. The cytotoxicity of CoPP, and ZnPP in NBL-6. The NBL-6 cells were treated with CoPP, and ZnPP at various concentrations (5, 10, 20, 40 and 80 μM) respectively for 24 h, the cytotoxicity of was detected by CCK-8.

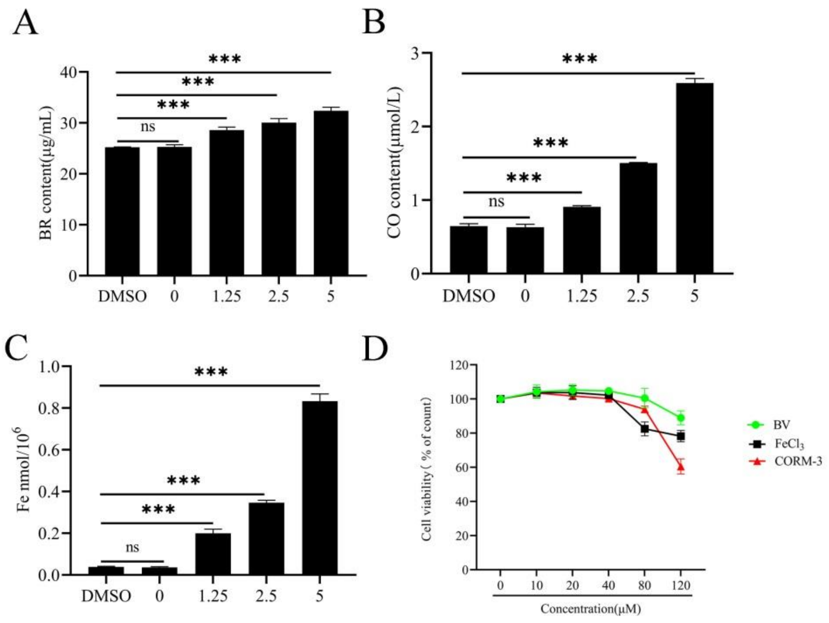

Figure S2. The BV, CO and iron concentrations detect by cepharanthine treatment, and the cytotoxicity of Biliverdin, $FeCl_3$, and CORM-3 in NBL-6. The NBL-6 cells were seeded into 6-well plates overnight, incubated with cepharanthine at various concentrations (1.25, 2.5 and 5 μM) or DMSO (control) for 2 h, infected 0.1

MOI EqHV-8 SDLC66 for 1 h. then, replaced with 3% FBS MEM containing indicated cepharanthine, and these cells were collected at 24hpi to test BV, CO and iron concentrations. The intracellular BR content was detected by ELISA (A), the CO content was detected by the endogenous CO detection kit (B), and the intracellular iron content was detected by Intracellular Iron Colorimetric Assay Kit (C). The NBL-6 cells were treated with Biliverdin, $FeCl_3$, and CORM-3 at various concentrations (10, 20, 40, 80 and 120 μM) respectively for 24 h, the cytotoxicity of was detected by CCK-8(D). Data are presented as the means of normalized data ± standard deviations (error bars) based on at least three independent experiments. *** $p < 0.001$; ns: no significant.

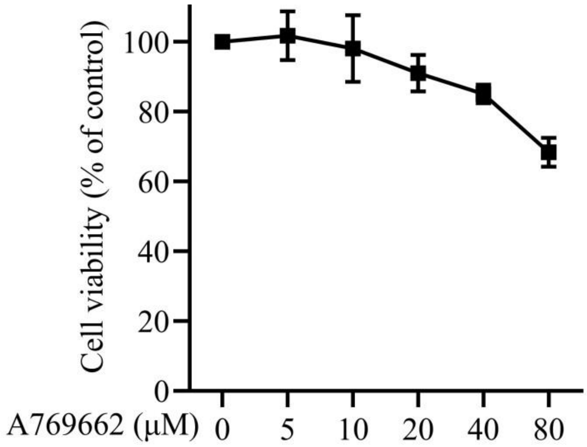

Figure S3. The A769662cytotoxicity detection in NBL-6. The NBL-6 cells were treated with A769662 at various concentrations (5, 10, 20, 40 and 80μM) respectively for 24 h, the cytotoxicity of was detected by CCK-8.

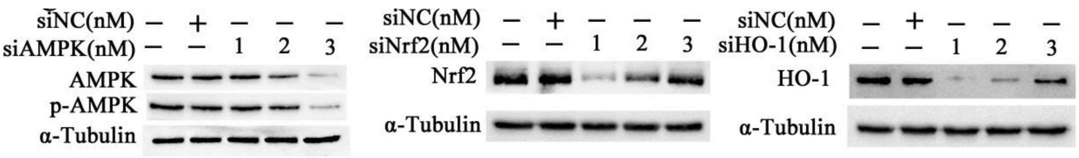

Figure S4. The effective siRNAs screening of AMPK, Nrf2 or HO-1 in NBL-6. The NBL-6 cells were seeded into 12-well plates and transfected with mixture of Lipo6000™ transfection reagent and siAMPK, siNrf2, siHO-1 or siNC (GenePharma, Shanghai, China) for 6h. Then the culture medium was replaced with 10% FBS MEM, these cells were collected at 48h to test AMPK, p-AMPK(A), Nrf2(B) and HO-1(C) protein expressions by Western blot.
